# New Appliances and Things Medical

**Published:** 1900-05-12

**Authors:** 


### NEW APPLIANCES AND THINGS MEDICAL.

**[We shall be glad to receive, at our Office, 28 & 29, Southampton Street, Strand, London, W.C., from the manufacturers, specimens of all new preparations and appliances which may be brought out from time to time.]**

### IZAL PREPARATIONS.

(Newton, Chambers, and Co., Limited, Thorncliffe, Near Sheffield.)

We have received samples of Izal liquid disinfectant, and that particular form of it which is intended for medical or internal use. We have also examined a specimen of Izal medical soap. Izal, as is well known, is a liquid emulsion of certain tar acids or phenols, combined with other bodies of antiseptic properties. As a germicide it has powers which have been widely recognised, and for the ordinary purposes of disinfcetion it is doubtless superior to carbolic acid, which has irritative and caustic properties, unless largely diluted and used with extreme care. Izal as an ordinary household disinfectant and antiseptic is pre-eminently safe and highly effective. The medical variety can be used in doses of three to five minims as an intestinal antiseptic. This form of Izal is particularly useful for gargles, enemata, and the like purpose. The Izal medical soap is not only to be recommended for surgical use and for disinfecting the skin, but, like other tar preparations, it has therapeutic properties of great merit in certain common diseases of the integument, and exercises on it a healthful influence even when affected with no such pathological complications.

### TOMATO KETCHUP.

(Gordon and Dilworth. Agents: Messrs. Fordham and Son, Limited, 36-40, York Road, King's Cross, London, N.)

We have received a sample bottle of the above condiment, which is specially prepared from fresh whole tomatoes by a new process ensuring purity and freedom from contamination. The finished product is piquant and delicious in flavour.

### PHENALGIN.

(Etna Chemical Company, New York, U.S.A.)

Phenalgin is a hypnotic having valuable properties, and chemically it is known as ammonio-phenyl-acetamide. Like other medicinal members of the anudo-benzene series, it has remarkable powers of assuaging the neuralgic pains accompanying rheumatism, influenza, and other constitutional conditions. For gastric neuralgias and all disturbances of the stomach it has exceptional efficacy, and acts particularly rapidly, owing, it is supposed, to the liberation of nascent ammonia, which further has a valuable stimulant action on the heart, a most desirable influence, contrasting favourably with the depressing action of certain closely allied bodies.

### PETANELLE DISINFECTING POWDER AND AUTOMATIC DISINFECTOR.

(Messrs. Paté, Burke, and Co., 6, Wool Exchange, Basinghall Street, London, E.C.)

The petanelle disinfecting powder consists of peat powder impregnated with solid and liquid deodorisers and antiseptics. The peat powder, being lighter than water, floats on the top of liquids in which it is scattered, and thereby counteracts or destroys offensive emanations given off from the surface. It may be used for the disinfection of solid matter in similar manner by sprinkling on the surface, and in this respect, for the deodorising of manure heaps, earth closets, ash-pits, and the like, it has valuable properties. It possesses a pleasant odour of turpentines, which is imparted to the surroundings, and altogether it constitutes an effective and pleasant deodorant for domestic or general use. The automatic disinfector, known as Walpole's, consists of a brass cylinder, containing a solid disinfectant which is soluble in water, and is intended to be placed in the supply cistern of water-closets and lavatories. Through small apertures in the cylinder a certain quantity of the disinfectant diffuses into the surrounding water, and each time the cistern is supplied passes into the pan or basin and disinfects the contents. The method is completely automatic, and a single charging of the cylinder is sufficient for many months. Owing to the oxidising properties of the disinfectant there is some risk of damage to iron fittings, and discoloration of the surface of china and earthenware pans or basins may follow in some cases.

